# Integration of High-Resolution Laser Displacement Sensors and 3D Printing for Structural Health Monitoring

**DOI:** 10.3390/s18010019

**Published:** 2017-12-22

**Authors:** Shu-Wei Chang, Tzu-Kang Lin, Shih-Yu Kuo, Ting-Hsuan Huang

**Affiliations:** 1 Department of Civil Engineering, National Taiwan University, No. 1, Sec. 4, Roosevelt Rd., Da’an Dist., Taipei 106, Taiwan; changsw@ntu.edu.tw (S.-W.C.); tlkfc8@gmail.com (S.-Y.K.); 2 Department of Civil Engineering, National Chiao Tung University, No. 1001, Daxue Rd., East Dist., Hsinchu 300, Taiwan; mn1875@gmail.com

**Keywords:** 3D printing, structural health monitoring, multiscale entropy, composites

## Abstract

This paper presents a novel experimental design for complex structural health monitoring (SHM) studies achieved by integrating 3D printing technologies, high-resolution laser displacement sensors, and multiscale entropy SHM theory. A seven-story structure with a variety of composite bracing systems was constructed using a dual-material 3D printer. A wireless Bluetooth vibration speaker was used to excite the ground floor of the structure, and high-resolution laser displacement sensors (1-μm resolution) were used to monitor the displacement history on different floors. Our results showed that the multiscale entropy SHM method could detect damage on the 3D-printed structures. The results of this study demonstrate that integrating 3D printing technologies and high-resolution laser displacement sensors enables the design of cheap, fast processing, complex, small-scale civil structures for future SHM studies. The novel experimental design proposed in this study provides a suitable platform for investigating the validity and sensitivity of SHM in different composite structures and damage conditions for real life applications in the future.

## 1. Introduction

In recent years, 3D printing technologies have emerged as a promising method for creating complex materials and structures. Such technologies not only provide an affordable means of creating complex models, but they also enable the design and construction of complex geometric objects [1,2,3]. Therefore, 3D printing can serve as a highly suitable manufacturing process for creating structures with complex geometries that are often difficult to fabricate in structural health monitoring (SHM) experiments.

Scholars have shown that 3D printing technologies enable the fabrication of complicated 3D microstructures with a broad range of materials. For example, polylactide (PLA), acrylonitrile butadiene styrene, and a wide range of polymer materials have been used extensively in low-cost 3D printers. Furthermore, an innovative selective laser sintering approach has been reported to have enabled the production of ceramics and metals with a resolution of less than 30 μm [4,5,6]. Another crucial feature of 3D printing technologies is the availability of 3D printers with multiple printing heads that can fabricate complicated composite structures [7,8]. In addition, bioinspired composite structures can be achieved by using a dual print head; for example, such structures can be realized by printing the main structure with a stiffer material and using a softer material, such as a cohesive or adhesive bond [9].

Despite recent developments in 3D printing technologies, controlling the geometry of objects is usually simple, whereas controlling their mechanical properties is difficult. However, recent studies have shown that the elastic properties of materials can be controlled by designing microstructures [10,11,12]. When the microstructures of a printed object are well designed, the elastic properties of a printed object of the same material can be controlled. This microstructural design approach provides an opportunity to fabricate a single structure with various mechanical properties by using the same material, and enables the easy fabrication of structures with desired properties by using currently available 3D printers.

The standard process of SHM can be categorized into four levels: detection of the presence of damage, assessment of the damage location, quantification of the damage, and prediction of service life [13]. Many recent studies have been devoted to SHM, which can be broadly divided into global health monitoring and local health monitoring. Visual inspection, the use of a strain gauge and an inclinometer are some typical ways applied for local health monitoring [14,15]. Over the past decades, a vibration-based SHM concept for assessing the condition of structures has emerged for global health monitoring [16], and diverse methods have been prosperously developed [17,18,19,20]. For example, the mode shape curvature and the natural frequency were utilized as an index to detect the possible damage [21,22]. In recent years, entropy analysis has been increasingly utilized in the field of SHM. In 2015, Lin and Liang proposed an SHM system based on multiscale cross-sample entropy (MSCE) [23]. Rojas et al. detected artificial damage on a plate-like structure by using short time wavelet entropy [24]. In 2017, Lin et al. developed an entropy-based damage detection method for 3D structures [25]. Considering the recent successful results, the entropy-based method was adopted in this study. Aerospace, civil, and marine structures are usually complex in terms of not only their geometries but also the design of their mechanical properties. However, most experiments consider simplified structures because of the high cost and difficulties of fabricating complex real structures. High-accuracy structural monitoring sensors are usually large and not applicable on 3D-printed structural models. Because of the light weight of 3D-printed structural models, the weights of commonly used structural monitoring sensors would significantly alter the dynamic responses of the original structures. Therefore, high-resolution non-contact monitoring sensors are required for monitoring 3D-printed structural models. High-resolution laser displacement sensors (1 μm) have been used extensively in the industry and are suitable monitoring sensors for small specimens such as 3D-printed small-scale specimens.

Although many damage detection algorithms based on measured vibration data are developed to detect possible damages in lab structures, the issue of how to conduct complex damage conditions in the experiments has always been a tough challenge. In this study, the idea is to utilize the advantage of high-resolution laser displacement sensors and precise 3D printing technology in damage simulation to demonstrate that a novel structural health monitoring system can be developed for studying complex damage conditions in SHM. We used 3D printing technologies to create a seven-story specimen and a variety of composite bracing systems to demonstrate the capability of realizing high-resolution complex structural models. A Bluetooth wireless vibration speaker was attached to the base of the 3D-printed structure to excite the structure. High-resolution laser displacement sensors (1-μm resolution) were used to monitor the displacement history of the different floors. A multiscale entropy SHM method was used to demonstrate that the novel experimental approach provides an effective platform for SHM studies for complex structures and composite materials under various damage conditions. This platform also serves as a systematic method of studying novel composite materials for civil engineering applications (e.g., novel lightweight composite materials for engineering applications) and investigating their damage.

## 2. Materials and Methods

### 2.1. The 3D-Printed Structural Model and Composite Bracing Systems

**Figure 1:**
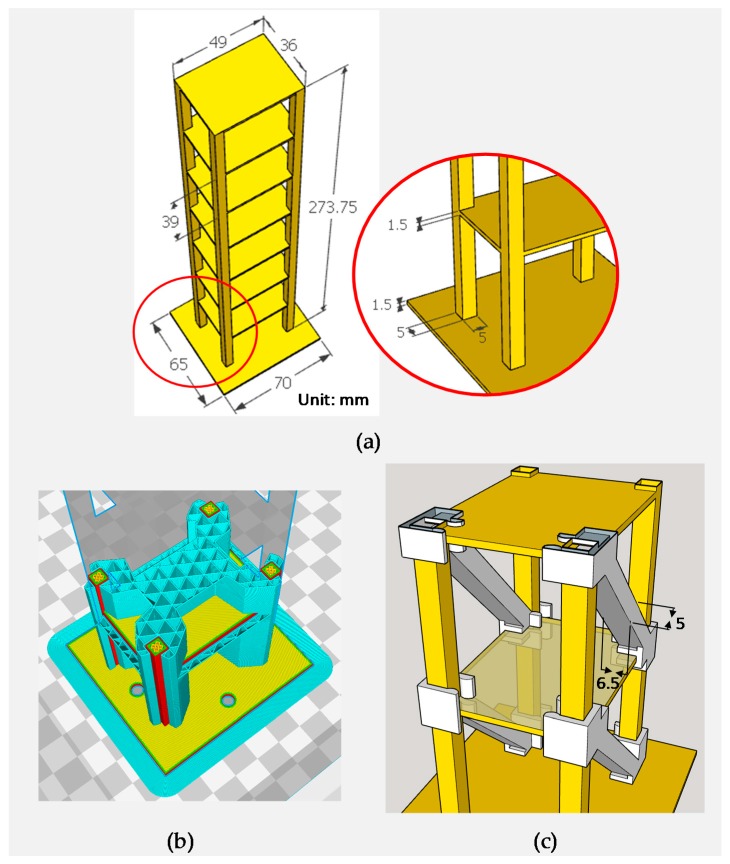
Dimension of the specimen and bracing systems. (**a**) 3D-printed seven-story specimen; (**b**) Design of the supporting material and slicing results; (**c**) Installation of the bracing systems.

A seven-story structure was designed based on an experimental model in a previous study [17]. The dimensions of the structure were based on a 1/30 model of the published model. The 3D structure was created using SketchUp 2016 (Trimble Inc., Sunnyvale, CA, USA). The dimensions of the scaled-down specimen are shown in Figure 1a. The height, length, and width of each story were 39, 49, and 36 mm, respectively. The thickness of the floor was 1.5 mm and the cross section of each column was 5 mm × 5 mm.

A dual-material 3D printer, Ultimaker 3 Extended (Ultimaker B.V., Geldermalsen, The Netherlands), with two 0.4 mm nozzles was used to print the seven-story structure and the bracing systems. The structure was printed using PLA. Supporting materials between floors were printed using polyvinyl alcohol (PVA). CURA 2.5 (Ultimaker B.V.) was used to slice the model. The structure of the supporting material and the slicing results are shown in Figure 1b. The primary settings for the printing process are presented in Table 1. After printing, the supporting material, namely PVA, was removed by submerging the structure in water. The adopted bracing system is shown in Figure 1c. A total of 14 PLA removable bracing systems were designed, as shown in Figure 1c. The cross section of each bracing was 6.5 mm × 5 mm. The bracing systems were installed symmetrically in the weak axis direction. In other words, each floor was installed with two bracing systems, as illustrated in Figure 1c, to represent the healthy condition of the structure.

**Figure 2:**
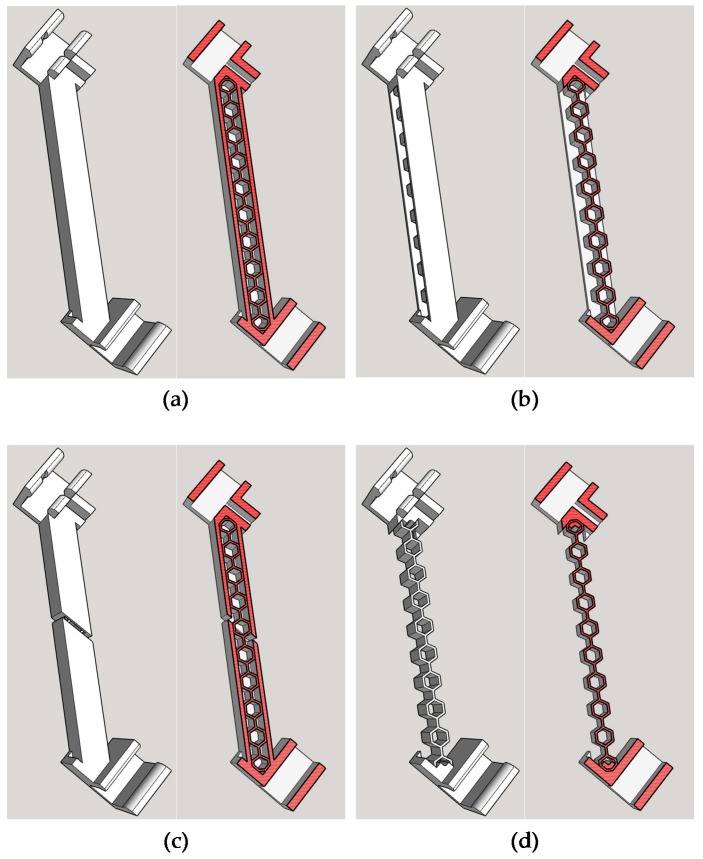
The designs of the composite bracing systems. (**a**) Honeycomb infill bracing; (**b**) Honeycomb sandwich bracing; (**c**) Damaged honeycomb infill bracing; (**d**) Honeycomb bracing.

As shown in Figure 2, the following four composite bracing systems were designed: (a) a honeycomb infill bracing; (b) a honeycomb sandwich bracing; (c) a damaged honeycomb infill bracing; and (d) a honeycomb bracing. Figure 2a presents the design of the honeycomb infill bracing, indicating that it comprised a PVA honeycomb infill structure covered with PLA layers. Figure 2b shows the honeycomb sandwich bracing. The damaged honeycomb infill bracing was designed to simulate a loss of stiffness caused by breaking the outer PLA layer, as illustrated in Figure 2c. The final bracing was constructed by removing the outer PLA layer of the honeycomb infill bracing, as shown in Figure 2d.

### 2.2. Experimental Setup

**Figure 3:**
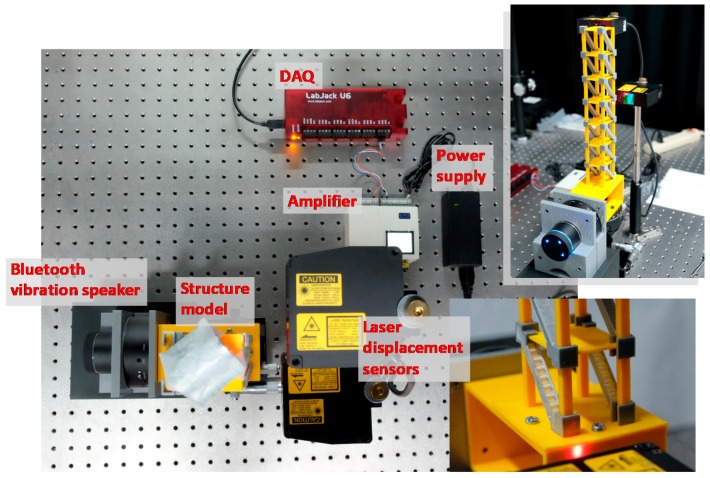
The experimental setup. The specimen was printed by the 3D printer. A Bluetooth vibration speaker was used to excite the specimen, and two laser displacement sensors were used to monitor the floor displacements. Various composite bracing systems were fabricated to simulate the various damage conditions of the specimen.

A Bluetooth vibration speaker (Newadin Technology Co. Ltd., Shenzhen, China) was used to excite the specimen. Two high-resolution laser displacement sensors (Optex Co. Ltd., Ogoto, Japan) were used to monitor the displacement history. The measuring range was 30 ± 5 mm, and the resolution was 1 μm. The data were transferred to a computer via an amplifier (Optex Co. Ltd., Ogoto, Japan) and a data acquisition (DAQ) system (LabJack Corporation, Lakewood, CO, USA), and they were collected using LabVIEW (National Instruments Corporation, Austin, TX, USA), as shown in Figure 3.

A 3D-printed foundation was designed to connect the structure and the vibration speaker. The seven-story structure was fixed on top of the foundation, with the vibration speaker attached to the left side of the foundation for the input of horizontal displacements. A mass of 202 g (±2.17 g) was placed on each floor to simulate the actual structural characteristics. Structural damage was simulated by removing the installed bracing systems from selected floors or by replacing the bracing on the first floor with composite bracing systems. In all experiments, white noise was the input to simulate the ambient vibration in the horizontal direction. In the experiments, 60 s of data were collected 1–2 min after the input of white noise. The displacement of the monitoring floor was determined with a sampling rate of 500 Hz. The measuring point of each floor was set on one column of the structure, at the height of the floor, as shown in the Figure 3.

### 2.3. The Entropy-Based SHM Algorithm

As the entropy-based method has been demonstrated to be more sensitive in detecting minor damage, compared to the traditional SHM method, it is adopted in this study. The multiscale concept was applied to convert an original vibration time history into signals at different time scales. First, a new time series $\left\{y_{j}^{\left(\tau \right)}\right\}$ can be established from the measured time series $x_{1},x_{2},\ldots ,x_{N}$ of length N where τ is the scale factor as follows:

(1) yj(τ)=1τ∑i=(j−1)τ+1jτxi, 1≤j≤Nτ

The similarity of a system can be quantified by calculating the entropy value of a measured time series. For the time series $y_{j}^{\left(\tau \right)}$, a vector of m data points can be defined as the template. The template space T of the signal represents the combination of all templates with length m. The time series may be composed of various N − m + 1 templates. Let $d_{ij}$ be the distance between two templates defined as $d_{ij}=max\left\{\left|x\left(i-k\right)-x\left(i+k\right)\right|:0\leq k\leq m-1\right\}$, and r be a predetermined threshold, the number of similarities $n_{i}^{m}\left(r\right)$ between templates $u_{m}\left(i\right)$ and $u_{m}(j)$ where $u_{m}\left(i\right)=\left\{x_{i},x_{i+1},\ldots ,x_{i+m-1},1\leq i\leq N-m+1\right\}$ can be calculated as follows:

(2) $$n_{i}^{m}\left(r\right)=\overset{N-m}{\underset{j=1}{\sum }}d\left[u_{m}\left(i\right){,u}_{m}\left(j\right)\right]$$

where

(3) $$d\left[u_{m}\left(i\right){,u}_{m}\left(j\right)\right]=\{\begin{array}{cc} 1 & d_{ij}\leq r\\ 0 & d_{ij}>r \end{array}$$

Other templates can be substituted for similarity comparisons with template i, and the average degree of similarity can be further calculated as follows:

(4) $$U^{m}\left(r\right)=\frac{1}{\left(N-m\right)}\sum_{i=1}^{N-m}U_{i}^{m}(r)$$

These steps are repeated to obtain the average degree of similarity $U^{m+1}\left(r\right)$ of the new template space, and the sample entropy values of the time series can be subsequently obtained as follows:

(5) $$S_{E}\left(m,r,N\right)=-\ln\frac{U^{m+1}\left(r\right)}{U^{m}\left(r\right)}$$

$S_{E}$ is the multiscale sample entropy (MSE) of the time series.

Cross-sample entropy (Cross-SampEn) is utilized to evaluate the degree of asynchrony or dissimilarity between two sensors from the same system. The similarity degree between templates $u_{m}\left(i\right)$ and $v_{m}(j)$ from different sensors is defined as $n_{i}^{m}\left(r\right)$ and is calculated under the following criterion:

(6) $$d\left[u_{m}\left(i\right){,v}_{m}\left(j\right)\right]\leq r,1\leq j\leq N-m$$

The average similarity probability of the template of length m can be calculated using the following equation:

(7) $$U^{m}\left(r\right)\left(v\|u\right)=\frac{1}{\left(N-m\right)}\sum_{i=1}^{N-m}U_{i}^{m}\left(r\right)\left(v\|u\right)$$

where $U^{m}\left(r\right)\left(v\|u\right)$ is the dissimilarity degree between the two time series with m points segmented.

By assembling templates with length m + 1, the average similarity probability $U^{m+1}\left(r\right)\left(v\|u\right)$ is used to derive the Cross-SampEn values as follows:

(8) $${\mathrm{CS}}_{E}\left(m,r,N\right)=-\ln\left\{\frac{U^{m+1}\left(r\right)\left(v\|u\right)}{U^{m}\left(r\right)\left(v\|u\right)}\right\}$$

A damage index (DI) is proposed, and the location of damage in a structure can be determined. For a structure with N stories, the MSCE of each story is expressed as the Cross-SampEn curve of the adjacent upper story to each story at a different scale. The MSCE curves for the healthy and damaged conditions of the structure are represented as H and D, respectively, and the subscripts depict the floor numbers; for example, $H_{1}$ is the MSCE between the ground and first floors of the healthy structure. The vertical characteristics of the first floor can be expressed as $H_{1}=\left\{{{{\mathrm{CS}}_{E}}^{1}}_{H_{1}},{{{\mathrm{CS}}_{E}}^{2}}_{H_{1}},{{{\mathrm{CS}}_{E}}^{3}}_{H_{1}},\cdots ,{{{\mathrm{CS}}_{E}}^{\tau }}_{H_{1}}\right\}$, where ${\mathrm{CS}}_{E}$ is the Cross-SampEn value, the superscript τ is the scale factor, and the subscript number indicates the evaluated floor. The MSCE of each floor of the damaged structure can then be expressed as follows:

(9) $$D_{F}=\left\{{{{\mathrm{CS}}_{E}}^{1}}_{D_{F}},{{{\mathrm{CS}}_{E}}^{2}}_{D_{F}}{{{\mathrm{CS}}_{E}}^{3}}_{D_{F}},\cdots ,{{{\mathrm{CS}}_{E}}^{\tau }}_{D_{F}}\right\}$$

The DI can be calculated as follows:

(10) $${\mathrm{DI}}_{F}=\overset{\tau }{\underset{q=1}{\sum }}\left({{{\mathrm{CS}}_{E}}^{q}}_{D_{F}}-{{{\mathrm{CS}}_{E}}^{q}}_{D_{F}}\right)$$

where F is the number of the floor for damage detection.

A positive DI value indicates the existence of damage on the floor as the involved structural damage causes an increase in dissimilarity in the individual structural responses, and a negative value indicates no damage on the floor.

## 3. Damage Condition by Removing Bracing

**Figure 4:**
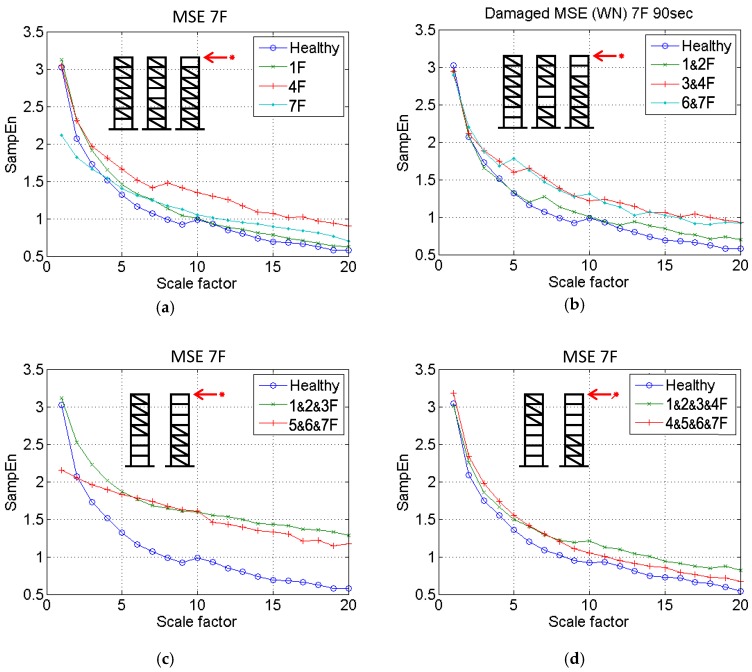
Multiscale sample entropy (MSE) diagrams of different damage categories. (**a**) one-story damage; (**b**) two-story damage; (**c**) three-story damage; (**d**) four-story damage.

The proposed algorithm was first applied to detect the damage condition of the scaled-down specimen by removing sold bracing which was the same damage scenario induced in the previous experimental study [19]. Ten damage cases, including single-story, two-story, three-story, and four-story damage, were examined on the 3D-printed model. In this study, the damage location was simulated as the loss of stiffness on a specific floor level. For example, the damage on the second floor is defined as the loss of stiffness between the second and third floor. The details are listed in Table 2. The vibration signal measured from the roof by using the high-resolution laser displacement sensor was processed through MSE analysis. The MSE distribution of each category is illustrated in Figure 4.

### 3.1. Single-Story Damage

As shown in Figure 4a, the distributions of the MSE curves for cases with damage on the first floor (1F), the fourth floor (4F), and the seventh floor (7F) are shown to be significantly higher than that of the curve for the healthy condition, which correlates strongly with the theoretical expectation. The curve for the 1F damage case is close to that for the 7F damage case, and the curve for the 4F damage case occupies the highest SampEn value. Because of the relatively slight impact of removing the bracing from a single story, the damage level is approximately reflected. However, the structure could still be reliably identified as damaged through MSE analysis.

### 3.2. Two-Story Damage

In the case of two-story damage, a similar trend to that of the single-story damage case was observed. The three damage curves (for the cases with damage on the first and second floors (1&2F), the third and fourth floors (3&4F), and the sixth and seventh floors (6&7F)) are shown to be higher, reflecting an increase in complexity from the vibration signal. It demonstrates that the structure was prone to instability due to the removal of the bracing. The 3&4F and 6&7F damage case curves are shown to deviate considerably from the reference healthy curve, thereby providing an easy method of detecting possible damage to the structure.

### 3.3. Three-Story Damage

A notable phenomenon can be observed in Figure 4c. The 1&2&3F and the 5&6&7F MSE curves for cases with damage on the first, second, and third floors (1&2&3F) and the fifth, sixth, and seventh floors (5&6&7F) are higher than for the healthy curve, indicating that the specimen exhibited a larger reduction in stiffness when compared with the healthy structure. Moreover, in contrast to the MSE values of the first two cases (single-story and second-story), the sequence of the MSE curves followed the physical condition with a higher distribution, indicating a more severe damage condition. The damage condition could be inferred based on the results.

### 3.4. Four-Story Damage

In the case of four-story damage, the MSE curve for cases with damage on the first, second, third, and fourth floors (1&2&3&4F) exhibits the highest peak, indicating that the corresponding four floors had the highest damage levels. The gap between the two curves (1&2&3&4F and 4&5&6&7F damage cases) is enlarged, indicating a major difference between damage occurring on the superstructure and on the substructure.

## 4. Damage Location

The 10 damage cases were further used to verify the feasibility of replicating the complicated laboratory or on-site experiments through the proposed SHM method.

### 4.1. Single-Story Damage

**Figure 5:**
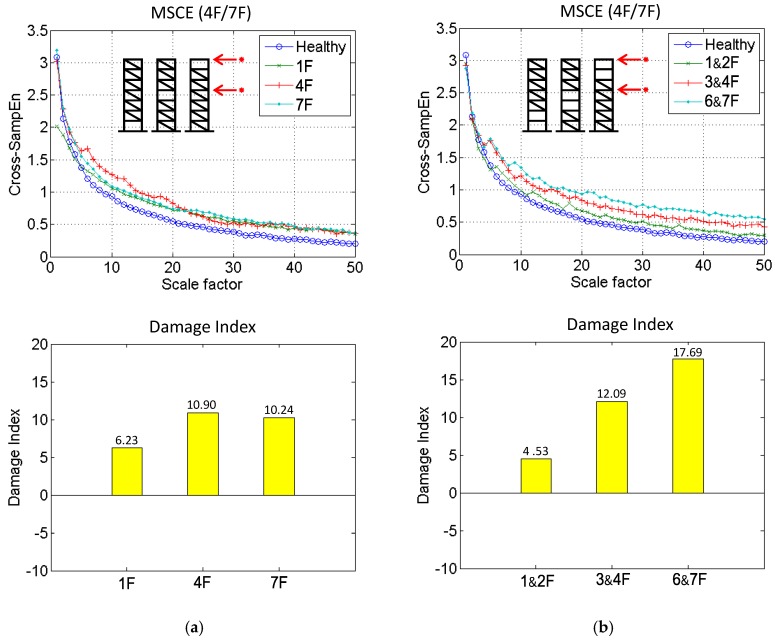
Multiscale cross-sample entropy (MSCE) and damage index (DI) diagrams of (**a**) single-story and (**b**) two-story damage.

In the case of single-story damage, as shown in Figure 5a, the time history of the displacement response of the sensors on the fourth floor (4F) and the seventh floor (7F) under white noise excitation was processed to detect whether the damage was located on the upper structure (above the 4F). The MSCE curves (4F/7F), illustrating the dissimilarity of the upper structure, are compared with the healthy case in Figure 5a, and the corresponding DI values are shown at the bottom of the figure. The MSCE curves are shown to deviate significantly from the blue reference curve. Moreover, the damage location can be indicated by the proposed DI. Compared with the DI value of 6.23 for the 1F damage case, the DI values for the 4F and the 7F damage cases are shown to be 10.90 and 10.24, respectively, both of which show the significant possibility of the damage being located in the upper structure.

### 4.2. Two-Story Damage

Three damage cases were simulated by removing the bracing from the two stories; thus, the damage was located on the 1&2F, 3&4F, and 6&7F. The MSCE diagrams for 4F/7F and the corresponding DI values are shown in Figure 5b. Due to the sensors being installed to monitor the response on the upper structure, the curves for the 3&4F and 6&7F damage cases are shown to fluctuate drastically, and the curve for 1&2F is distributed similarly to the curve for the healthy case. The DI value is revealed to have increased from 4.53 (1&2F damage case) to 12.09 (3&4F damage case), and then to 17.69 (6&7F damage case) as the damage location shifts closer to the sensor on the upper structure. Hence, the damage location can be reliably reflected by the DI.

### 4.3. Three-Story Damage

**Figure 6:**
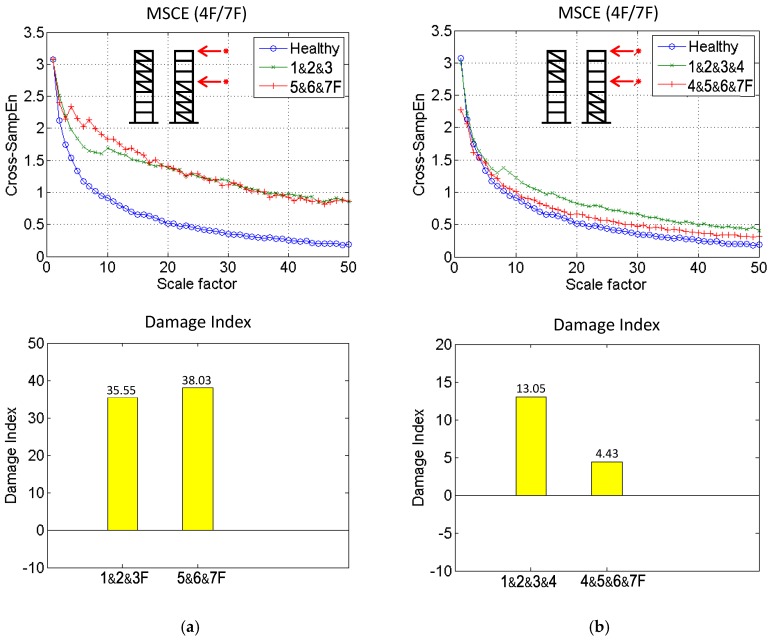
MSCE and DI diagrams of (**a**) three-story and (**b**) four-story damage.

To thoroughly examine the proposed method, two damage cases were further simulated by removing the bracing from three stories; thus, the damage was located on the 1&2&3F and the 5&6&7F. The MSCE diagrams for 4F/7F and the corresponding DI values are illustrated in Figure 6a. The two curves are shown to fluctuate drastically above that of the healthy case, indicating serious damage to the structure. The DI values for the 1&2&3F and the 5&6&7F damage cases are shown to be 35.55 and 38.03, demonstrating that the structure has been severely damaged.

### 4.4. Four-Story Damage

The most severe damage cases were simulated by removing the bracing from four stories; thus, the damage was located on 1&2&3&4F and 4&5&6&7F. The MSCE diagrams for 4F/7F and the corresponding DI values are depicted in Figure 6b. In contrast to Figure 6a, in which both curves fluctuate drastically above that of the healthy case, the MSCE curves are located slightly above the healthy curve. Similarly, the DI values for the 1&2&3&4F and 4&5&6&7F damage cases are shown to be 13.05 and 4.43, respectively. A possible reason for this phenomenon may be that the introduction of damage on four separate stories completely changed the global structural behavior.

## 5. Damage Degree (Composite)

**Figure 7:**
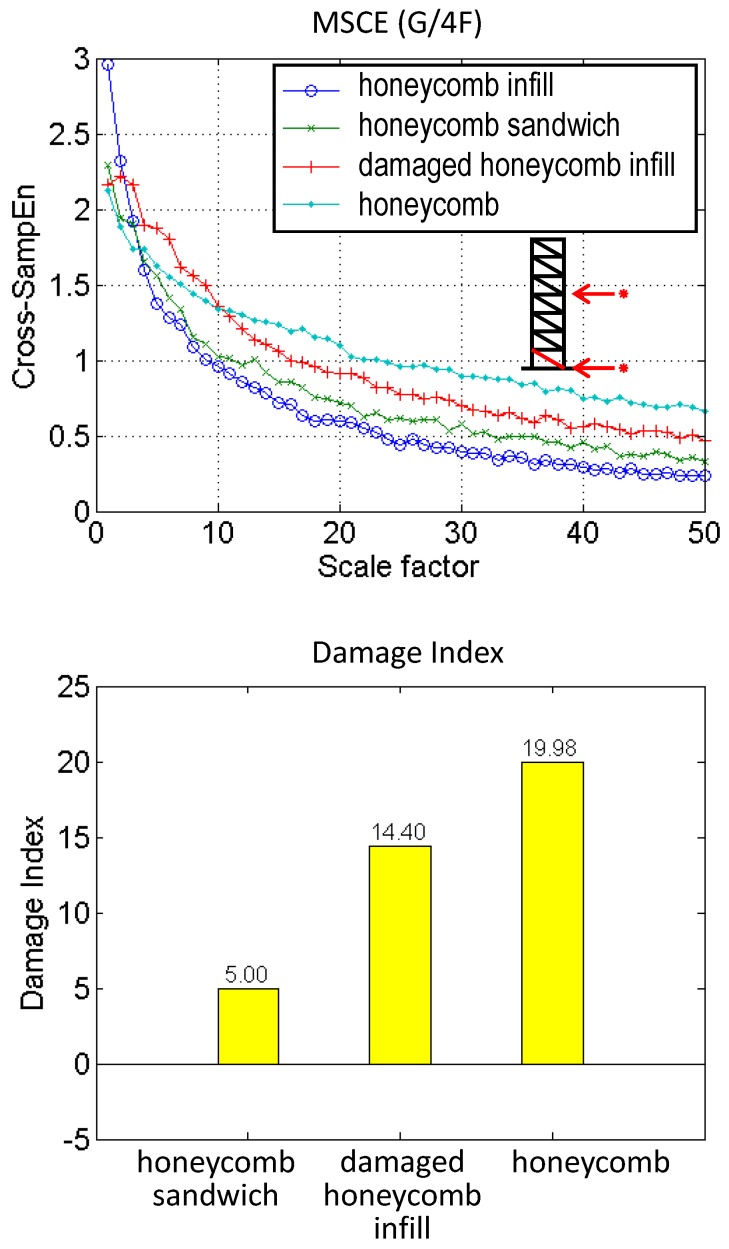
MSCE and DI diagrams of composite structure.

The degree of damage was examined using the composite material as the bracing element. Four composite structures were introduced: the honeycomb infill bracing, the honeycomb sandwich bracing, the damaged honeycomb infill bracing, and the honeycomb bracing. In contrast to the cases described in the previous section, sensors were deployed on the ground and fourth floors. The damage was simulated by replacing the two bracing systems on the first story with composite bracings, and the case of the honeycomb infill bracing was treated as the reference case for damage degree detection. The MSCE curve (G/4F) and the corresponding DI values are shown in Figure 7. The MSCE curve of honeycomb infill bracing is shown to be distributed at the bottom. With an increase in the damage degree, the curves deviate to the upper part of the figure. Regarding the damage degree, the DI values for the cases involving the honeycomb sandwich, the damaged honeycomb infill bracing, and the honeycomb bracing are shown to be 5.00, 14.40, and 19.98, respectively. The results demonstrate that the degree of damage could be estimated by the proposed DI.

## 6. Discussion and Conclusions

In this paper, 3D printing technologies are demonstrated to provide a fast and low-cost fabrication method for creating complicated composite structures. A seven-story specimen with various types of composite bracing was printed for SHM experiments in this study. High-resolution (1 μm) laser displacement sensors enabled the monitoring of the dynamics of the specimen under white noise excitation from a Bluetooth vibration speaker. The results reveal that the MSE derived from single laser displacement monitoring on the uppermost story could distinguish the damage of the specimen. Furthermore, the MSCE derived from the fourth story and the ground floor could specify the damage in the substructure of the specimen. This experimental setup provides a novel platform for future fundamental SHM studies using 3D printing technologies. We believe that the integration of 3D printing technologies into SHM studies could advance the understanding of existing SHM theories. For example, it is possible to reconstruct a 3D image of a real damaged structure in real life applications and fabricate the reconstructed model by using 3D printing technologies in order to investigate the accuracy and performance of existing SHM theories.

## Figures and Tables

**Table 1:** Primary settings for 3D printing.

Materials	Model		Bracing	Composite Bracing	
	Polylactide (PLA) (Model)	Polyvinyl Alcohol (PVA) (Support)	PLA	PLA	PVA
Printing temperature (°C)	200	215	200	200	215
Bed temperature (°C)	60	60	60	60	60
Printing speed (mm/s)	45	32	50	50	15
Infill rate (%)	100	25	20	20	100
Layer thickness (mm)	0.1	0.1	0.1	0.1	0.1

**Table 2:** Damage cases.

Case Number	Damage Category	Damage Floors
1	Undamaged	None
2	Single-story	1F
3		4F
4		7F
5	Two-story	1 and 2F
6		3 and 4F
7		6 and 7F
8	Three-story	1, 2, and 3F
9		5, 6, and 7F
10	Four-story	1, 2, 3 and 4F
11		4, 5, 6 and 7F
